# Associations of exclusive e-cigarette use and dual use of e-cigarettes and combustible cigarettes with prevalent and incident hypertension: A systematic review and meta-analysis

**DOI:** 10.1016/j.ajpc.2026.101767

**Published:** 2026-07-24

**Authors:** Yusuff Adebayo Adebisi, Massimo Volpe, Jacob George, Ahmed K. Shukri, Najim Z. Alshahrani, Davide Capodanno, Bader Ali Almustafa, Anas Ali Alhur, Henry Eziefule Nwankwo, Deborah Temitope Adeyemo, Eseosa Iyagbaye, Duaa Abdullah Bafail, Riccardo Polosa

**Affiliations:** a College of Social Sciences, University of Glasgow, 40 Bute Gardens, Glasgow, G12 8RT, Scotland, United Kingdom; b Department of Clinical and Experimental Medicine, University of Catania, Catania, Italy; c Faculty of Pharmacy, University of Ibadan, Ibadan, Nigeria; d Hypertension and Cardiovascular Prevention Unit, IRCCS San Raffaele Roma, Rome, Italy; e Department of Clinical and Molecular Medicine, Sapienza University of Rome, Rome, Italy; f University of Dundee Medical School, Dundee, Scotland, United Kingdom; g Department of Family and Community Medicine, Faculty of Medicine, University of Jeddah, Jeddah, Saudi Arabia; h Division of Cardiology, AOU Policlinico G. Rodolico–San Marco, University of Catania, Catania, Italy; i Secretary General, World Hypertension League, Riyadh, Saudi Arabia; j Head, Scientific Committee, Saudi Hypertension Management Society, Riyadh, Saudi Arabia; k Department of Health Informatics, College of Public Health and Health Informatics, University of Hail, Hail, Saudi Arabia; l Department of Clinical Sciences and Nutrition, Faculty of Medicine, Dentistry and Life Sciences, University of Chester, Exton Park (Parkgate Road) Campus, Chester, CH1 4BJ, United Kingdom; m Faculty of Pharmaceutical Sciences, University of Ilorin, Ilorin, Nigeria; n Chronic Disease Mate for Africa, Benin City, Nigeria; o Department of Clinical Pharmacology, Faculty of Medicine, King Abdulaziz University, Jeddah, Saudi Arabia; p Center of Excellence for the Acceleration of Harm Reduction, University of Catania, Catania, Italy

**Keywords:** Electronic cigarettes, Vaping, Hypertension, Blood pressure, Dual use, Tobacco smoking, Meta-analysis, Systematic review

## Abstract

**Introduction:**

The relationship between electronic cigarette (e-cigarette) use and hypertension remains uncertain. Existing evidence is difficult to interpret because of differences in exposure classification, smoking history, dual use, study design, and hypertension ascertainment. We systematically reviewed and meta-analysed population-based observational studies examining associations between exclusive e-cigarette use, dual use of e-cigarettes and combustible cigarettes, and prevalent and incident hypertension.

**Methods:**

Following PRISMA, we searched PubMed/MEDLINE, Embase, Scopus, and Web of Science from inception to 31 May 2026. Eligible cross-sectional or longitudinal observational studies reported exposure-specific associations with prevalent or incident hypertension. Exclusive e-cigarette use was defined as current or recent e-cigarette use without concurrent combustible-cigarette use, and dual use as concurrent use of both products. Hypertension was ascertained through self-reported or clinician diagnosis, electronic health records, antihypertensive medication use, or a study-defined measured blood-pressure threshold. Acute experiments, studies reporting only continuous blood-pressure outcomes, interventional switching studies, and reports without extractable estimates were excluded. Adjusted estimates were pooled using random-effects restricted maximum likelihood models. Risk of bias was assessed using ROBINS-E, and certainty of evidence was evaluated using GRADE.

**Results:**

Of 3988 records identified, 10 studies were included: six cross-sectional or pooled cross-sectional studies and four longitudinal studies. For prevalent hypertension, the pooled estimate for exclusive e-cigarette use included the null (OR 1.09, 95% CI 0.88–1.36; I² = 68.1%), whereas dual use was associated with higher odds of hypertension (OR 1.41, 95% CI 1.16–1.72; I² = 80.9%). Three non-overlapping longitudinal cohorts contributed to the primary incident analyses, with reported follow-up ranging from 3.7 to 6.0 years. For incident hypertension, the estimate for exclusive e-cigarette use also included the null (HR 1.17, 95% CI 0.83–1.64; I² = 64.3%), whereas dual use was associated with a higher risk of incident hypertension (HR 1.35, 95% CI 1.03–1.76; I² = 77.3%). In separate secondary analyses, cigarette-only smoking was associated with prevalent hypertension (OR 1.25, 95% CI 1.10–1.41) and incident hypertension (HR 1.20, 95% CI 1.15–1.25). Certainty of evidence was very low because of risk of bias, heterogeneity, and imprecision.

**Conclusions:**

No statistically clear association was observed between exclusive e-cigarette use and prevalent or incident hypertension; however, the very low-certainty and imprecise evidence does not exclude a potentially important association. Dual use was associated with higher odds of prevalent hypertension and a higher risk of incident hypertension, although causal interpretation is limited by observational study designs, heterogeneity, residual confounding, and exposure misclassification.

## Graphical abstract

**Figure:**
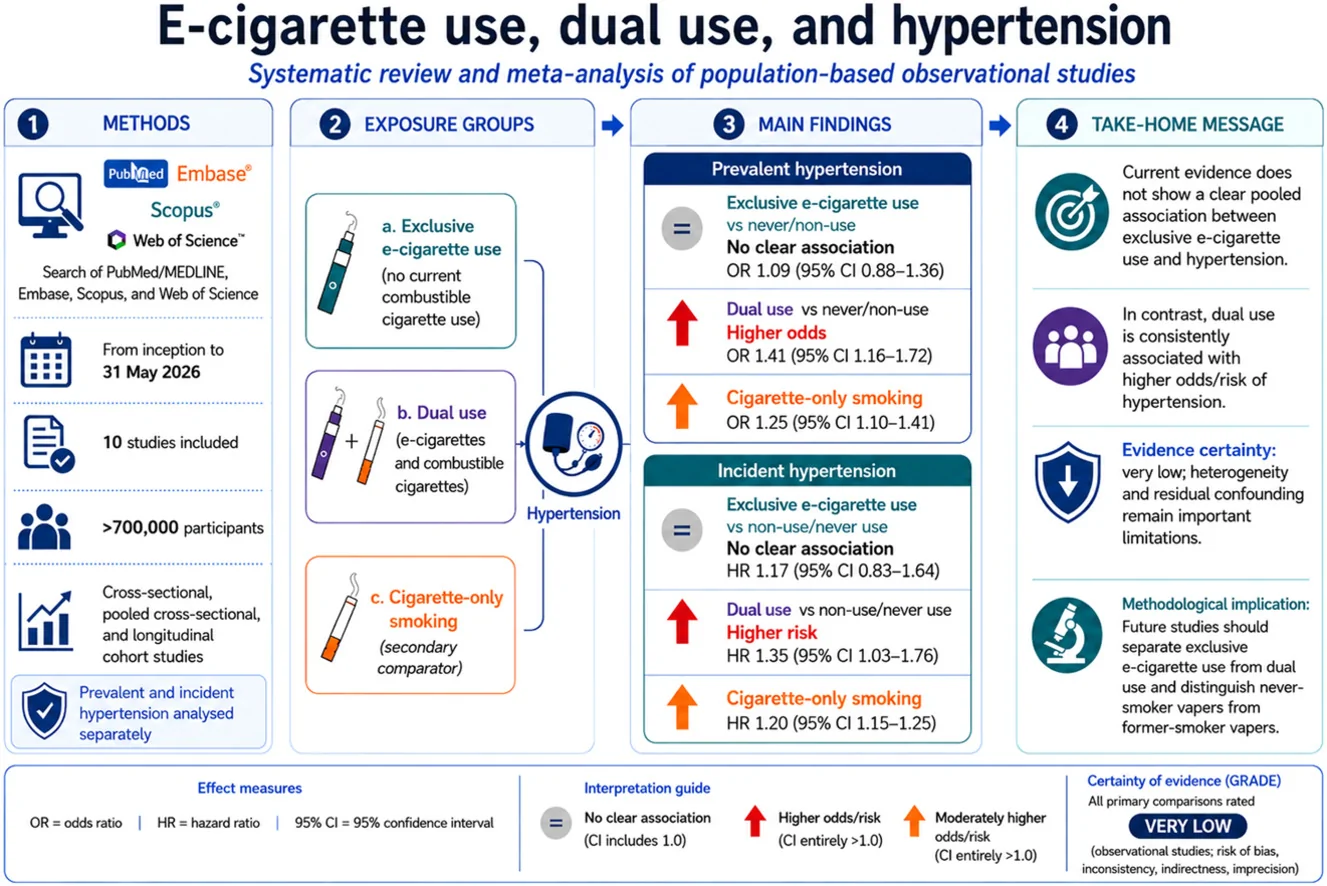

## 1 Introduction

Hypertension is the leading modifiable risk factor for cardiovascular morbidity and premature mortality worldwide, affecting more than one billion adults and contributing substantially to the global burden of stroke, ischaemic heart disease, heart failure, and chronic kidney disease [1,2]. Identifying and quantifying preventable contributors to hypertension therefore remains a major public-health priority. Combustible cigarette smoking is an established cardiovascular risk factor, but its relationship with measured or diagnosed hypertension in observational studies is more complex than is often assumed. Smokers often have lower body weight, acute nicotine exposure has transient pressor effects that may not translate into sustained hypertension, and cessation, reverse causation, and confounding by health behaviours can distort cross-sectional associations [3].

Electronic cigarettes, also known as e-cigarettes or electronic nicotine delivery systems, add further complexity. Experimental studies indicate that nicotine-containing e-cigarettes can acutely increase blood pressure and heart rate and impair endothelial function [[4], [5], [6]]. These short-term haemodynamic responses do not establish whether habitual use causes sustained, diagnosed, or measured hypertension at the population level [7,8]. Potential pathways relevant to chronic blood-pressure regulation include sympathetic activation and autonomic imbalance, oxidative stress, inflammation, endothelial dysfunction, arterial stiffness, and vascular remodelling. The magnitude of these effects may vary according to nicotine content, device characteristics, and non-nicotine aerosol constituents. Combustible cigarettes additionally expose users to combustion-derived toxicants, so the vascular exposure profile of cigarette-only and dual use differs materially from that of exclusive e-cigarette use.

Interpretation also depends on smoking history. Some people who use e-cigarettes have never smoked, some add e-cigarettes while continuing to smoke, and many switch after years of combustible-cigarette exposure. These groups differ in cumulative smoking, baseline cardiovascular risk, and reasons for product use. Population-based studies have consequently reported mixed findings [[9], [10], [11]], partly because exposure categories vary across current, former, ever, exclusive, dual, and switching use, and because hypertension has been ascertained by self-report, clinical measurement, medication use, or electronic health records. Previous reviews have largely addressed acute blood-pressure responses, vascular markers, or broad cardiovascular outcomes rather than hypertension as a defined population-based outcome separated by exposure category and outcome timing [5,10,11].

To our knowledge, this is the first systematic review and meta-analysis to separately evaluate exclusive e-cigarette use and dual use in relation to prevalent and incident hypertension. We also treated cigarette-only smoking as a secondary comparator and examined former smokers who currently used e-cigarettes as a distinct subgroup because of their prior combustible-cigarette exposure.

## 2 Methods

### 2.1 Reporting framework, protocol, and eligibility criteria

The review was conducted and reported in accordance with the Preferred Reporting Items for Systematic Reviews and Meta-Analyses (PRISMA) 2020 statement [12] and the Meta-analysis of Observational Studies in Epidemiology (MOOSE) guidance [13].

Studies were eligible if they were observational, including cross-sectional, pooled cross-sectional, or longitudinal cohort studies, and reported an association between e-cigarette use or dual use of e-cigarettes and combustible cigarettes and hypertension in a defined adult or general population. Cigarette-only smoking was extracted as a secondary comparator exposure. Hypertension was eligible when explicitly defined by self-report, doctor or other health-professional diagnosis, electronic health record diagnosis, antihypertensive medication use, or a study-defined measured blood-pressure threshold. For measured outcomes, the original investigators had to classify participants as hypertensive using their stated survey or clinical measurement protocol, typically systolic blood pressure ≥140 mmHg or diastolic blood pressure ≥90 mmHg. A single elevated reading without an explicit dichotomous hypertension classification was not sufficient; no universal number of measurement occasions was imposed because the included studies used different established protocols, which were extracted and reported at study level.

Studies were excluded if they reported only acute blood-pressure responses; blood pressure solely as a continuous outcome, such as a mean change in systolic or diastolic pressure; elevated blood pressure without an explicit hypertension definition; a metabolic-syndrome blood-pressure component; biomarker-only outcomes; interventional switching or blood-pressure-controlled trials; or no extractable exposure-specific hypertension estimate.

### 2.2 Information sources and search strategy

We searched PubMed/MEDLINE, Embase, Scopus, and Web of Science from database inception to 31 May 2026. These complementary biomedical and multidisciplinary databases were selected to provide broad coverage of observational epidemiological research. Database-specific searches combined controlled vocabulary, where available, with free-text terms for e-cigarettes, vaping, electronic nicotine delivery systems, hypertension, high blood pressure, and blood pressure. Backward and forward citation screening of included studies and relevant reviews supplemented the electronic searches. The complete reproducible search strings and search dates are provided in Supplementary Table S1.

### 2.3 Study selection and data extraction

Titles, abstracts, and full texts were screened independently against the eligibility criteria by two investigators, with disagreements resolved through discussion and consensus. The purpose of duplicate screening was to minimise erroneous exclusions; a formal chance-corrected agreement statistic was not prespecified or calculated. For each eligible study, two investigators independently extracted author, year, country, data source, design and period, population, exposure categories, comparator, hypertension definition, follow-up where applicable, covariate adjustment, effect measure, point estimate, 95% confidence interval (CI), and eligibility for primary pooling [14,15]. All author-reported estimates were retained in the extraction, but only one eligible estimate per study or non-overlapping cohort was selected for each pooled comparison. The complete extraction, including estimates not used in primary pooling, is presented in Supplementary Tables S2 and S3.

### 2.4 Outcomes and comparisons

The co-primary outcomes were prevalent hypertension, defined as hypertension present at the same assessment at which exposure was classified, and incident hypertension, defined as a new study-defined diagnosis or onset during follow-up among participants without hypertension at baseline. Exclusive e-cigarette use was defined as current or recent e-cigarette use without concurrent combustible-cigarette use according to the original study; it did not necessarily imply that the participant had never smoked. Dual use was defined as concurrent current or recent use of e-cigarettes and combustible cigarettes. The four primary comparisons were exclusive e-cigarette use versus never or non-use and dual use versus never or non-use, each analysed separately for prevalent and incident hypertension. Cigarette-only smoking was analysed as a secondary comparator. Where compatible estimates were reported, former smokers who currently used e-cigarettes were examined separately. Further stratification of exclusive users into never-smokers and former smokers was not undertaken because too few studies reported these categories consistently for a valid pooled analysis.

### 2.5 Handling of overlapping cohorts

Overlapping cohorts arose when separate publications analysed the same underlying participants at different survey waves or using alternative exposure definitions or models. Pooling such estimates together would give the same participants disproportionate weight and produce artificially precise confidence intervals. Therefore, one estimate was retained in the primary analysis for each cohort, exposure, and outcome comparison. Selection prioritised the closest match to the predefined exposure and outcome, the longer relevant follow-up, and the most complete adjusted reporting. For overlapping longitudinal analyses from the same cohort, the study reporting overall exposure estimates across the longer follow-up period was retained in the primary analysis, while alternative sex-stratified estimates were used in sensitivity analyses. Overlapping estimates were never pooled together in the same analysis.

Mutually exclusive subgroup estimates, such as separate estimates for male and female participants, were eligible when no overall estimate was available because the participants were not double-counted. Results were therefore reported as both numbers of estimates and numbers of studies. When one study reported several exposure definitions, such as ever, former, and current e-cigarette use, the estimate most closely matching current non-dual e-cigarette use was selected for the relevant primary comparison. This rule maintained exposure consistency while preventing multiple estimates from the same participants entering one pooled analysis.

### 2.6 Risk of bias and certainty of evidence

Risk of bias was assessed using the Risk of Bias In Non-randomized Studies of Exposure (ROBINS-E) tool [16]. Certainty of evidence was rated separately for each exposure-outcome comparison using the Grading of Recommendations Assessment, Development and Evaluation (GRADE) framework, considering risk of bias, inconsistency, indirectness, imprecision, and publication or reporting bias [17]. Because all included studies were observational, certainty began at low and was downgraded when serious concerns were identified. Publication bias was not formally assessed because each pooled comparison contained fewer than ten estimates.

### 2.7 Statistical analysis

Author-reported adjusted estimates were used preferentially. Odds ratios were pooled for prevalent hypertension and hazard ratios for incident hypertension. Estimates were transformed to the natural-logarithm scale, and standard errors were derived from the reported 95% confidence intervals. Random-effects meta-analysis was performed using restricted maximum likelihood estimation of between-study variance, and pooled estimates were back-transformed by exponentiation [14]. Statistical heterogeneity was quantified using τ² and I², with I² values of approximately 25%, 50%, and 75% interpreted as low, moderate, and substantial heterogeneity. A single pooled estimate across all studies was not calculated because outcome timing, effect measures, exposure definitions, comparator groups, and hypertension ascertainment were not sufficiently compatible. Funnel plots and Egger tests were not performed because all pooled comparisons contained fewer than ten estimates, for which such tests are underpowered and unreliable [15]. Analyses were conducted in StataNow 19.5.

## 3 Results

### 3.1 Study selection

The searches identified 3988 records: 671 from PubMed/MEDLINE, 2663 from Embase, 471 from Scopus, and 183 from Web of Science Core Collection. After 1762 duplicates were removed, 2226 titles and abstracts were screened. Of these, 2172 were excluded because they did not meet the review criteria, including records with ineligible study designs, no relevant e-cigarette or dual-use exposure, no defined hypertension outcome, acute or experimental blood-pressure assessments, continuous blood-pressure-only outcomes, non-human or laboratory research, non-original articles, conference abstracts, or otherwise irrelevant topics. Fifty-four reports underwent full-text assessment, of which 44 were excluded because they were not eligible population-based observational hypertension studies, reported only acute or continuous blood-pressure outcomes, lacked extractable exposure-specific estimates, used ineligible exposure or outcome definitions, or were reviews, editorials, conference abstracts, or overlapping reports. Ten studies were included in the qualitative synthesis and, where appropriate, the meta-analyses [[18], [19], [20], [21], [22], [23], [24], [25], [26], [27]]. More than 700,000 participants were represented overall, although two PATH publications used partially overlapping samples. The study-selection process is shown in Fig. 1.

**Fig. 1:**
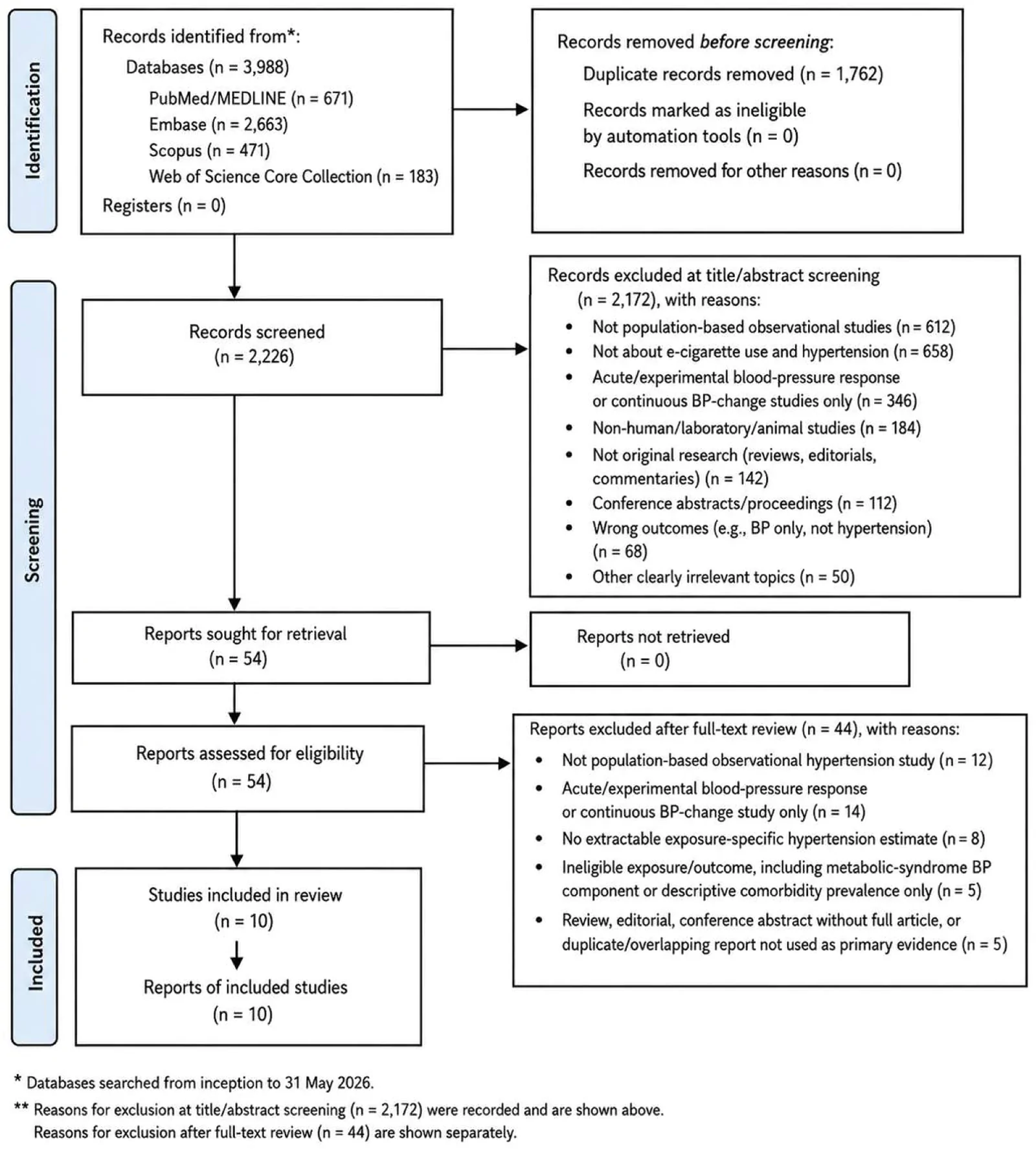
PRISMA 2020 flow diagram of study identification, screening, and inclusion.

### 3.2 Characteristics of included studies

The ten included studies were conducted in the United States [18,19,21,[22], [23], [24], [25],^27^], South Korea [20], and Scotland [26]. Five were cross-sectional [[18], [19], [20],^23^,^27^], one was pooled cross-sectional [26], and four were longitudinal cohorts [21,22,24,25]. Reported longitudinal follow-up was approximately 3.7–6.0 years. Hypertension was defined by self-report or doctor diagnosis [18,19,21,22,26], EHR-recorded incident diagnosis [24], antihypertensive medication use [23,25], measured blood-pressure thresholds [20,23,25,27], or combinations of these methods. Six studies contributed to prevalent-hypertension meta-analyses [[18], [19], [20],^23^,^26^,^27^], and three non-overlapping longitudinal studies contributed to the primary incident-hypertension analyses [22,24,25]. Shi et al. [21] was retained for sensitivity analysis because its PATH sample overlapped with Cook et al. [22]. Study characteristics are summarised in Table 1; detailed exposure definitions and adjustment are in Supplementary Table S2, all extracted estimates in Supplementary Table S3, and estimates used in primary pooling in Supplementary Table S4.

**Table 1:** Characteristics of the ten included studies.

Study (country)	Data source / design	Population	Hypertension outcome	Exposure categories	Contribution to synthesis
Miller 2021 (USA) [18]	PATH Wave 3; cross-sectional, 2015–2016	Adults 18–54 y (n = 19,147)	Self-reported diagnosed hypertension	Current vaping/smoking; six-category vaping/smoking status; dual use	Primary prevalent dual-use analysis; secondary analyses for cigarette-only smoking and former-smoker/current-vaper subgroup; direct e-cigarette vs cigarette-smoking comparisons extracted narratively
Falk 2022 (USA) [19]	NHIS 2014/2016/2017/2018; cross-sectional	Adults ≥18 y (n = 84,553)	Self-reported doctor/health-professional diagnosis	E-cig only, cigarette only, dual use, former-smoker/current-vaper	Primary prevalent exclusive e-cigarette and dual-use analyses; secondary analyses for cigarette-only smoking and former-smoker/current-vaper subgroup
Kim 2022 (S. Korea) [20]	Korea Community Health Survey 2019; cross-sectional	Adults ≥19 y; medication users excluded (n = 275,762)	Measured: SBP ≥140 or DBP ≥90 mmHg	E-cig only, cigarette only, dual smoker, past smoker (sex-stratified)	Primary prevalent exclusive e-cigarette and dual-use analyses; secondary cigarette-only smoking analysis
Shi 2023 (USA) [21]	PATH Waves 1–4; prospective cohort	Adults without lifetime hypertension at baseline (n = 16,434)	Self-reported incident diagnosis	Exclusive e-cig, exclusive cigarette, dual use (sex-stratified)	Sensitivity analysis only because of overlap with another PATH longitudinal study; used for sex-stratified incident estimates
Cook 2023 (USA) [22]	PATH Waves 1–5; prospective cohort	Adults free of hypertension/CVD at baseline (n = 17,539)	Self-reported incident diagnosis	Non-use, exclusive cigarette, exclusive ENDS, dual use	Primary incident exclusive e-cigarette and dual-use analyses; secondary incident cigarette-only smoking analysis
Lu 2024 (USA) [23]	NHANES 2015–2018; cross-sectional	Adults ≥20 y with complete data (n = 7696)	Measured BP, self-report, or antihypertensive medication	E-cig only, cigarette only, dual use	Primary prevalent exclusive e-cigarette and dual-use analyses; secondary cigarette-only smoking analysis
Erhabor 2025 (USA) [24]	All of Us; longitudinal, EHR-linked	Adults with linked EHR data (n = 249,190)	Incident EHR-recorded hypertension	Non-use, exclusive e-cig, exclusive cigarette, dual use	Primary incident exclusive e-cigarette and dual-use analyses; secondary incident cigarette-only smoking analysis
Lee 2026 (USA) [25]	HCHS/SOL; population-based cohort	Hispanic/Latino adults 18–74 y (n = 11,593 prevalent; 6306 incident)	Measured BP ≥140/90 mmHg or antihypertensive treatment	Ever/former/current e-cig; dual ever use	Primary incident exclusive e-cigarette and dual-use analyses; prevalent prevalence-ratio estimates extracted but not pooled with odds ratios
Adebisi 2026 (Scotland) [26]	Scottish Health Survey 2017–2022; pooled cross-sectional	Adults ≥16 y in private households (n = 22,187)	Self-reported doctor-diagnosed high blood pressure	Never, former smoker, cigarette only, e-cig only, dual use	Primary prevalent exclusive e-cigarette and dual-use analyses; secondary analyses for cigarette-only smoking and former-smoker/current-vaper subgroup; direct e-cigarette vs cigarette-smoking comparison extracted narratively
Corsi & Agbaje 2026 (USA) [27]	NHANES 2021–2023; cross-sectional	Participants ≥12 y with complete status (n = 6262)	Measured: SBP ≥140 or DBP ≥90 mmHg	Exclusive smoker, exclusive vaper, dual user	Primary prevalent exclusive e-cigarette and dual-use analyses; analytically distinct because of measured hypertension outcome and recent smoking/vaping exposure window

PATH Population Assessment of Tobacco and Health; NHIS National Health Interview Survey; NHANES National Health and Nutrition Examination Survey; HCHS/SOL Hispanic Community Health Study/Study of Latinos; EHR electronic health record; ENDS electronic nicotine delivery systems; CVD cardiovascular disease; SBP systolic blood pressure; DBP diastolic blood pressure.

### 3.3 Primary meta-analyses

The primary pooled results are displayed in Fig. 2, Fig. 3, Fig. 4, Fig. 5. For prevalent hypertension, the estimate for exclusive e-cigarette use versus never or non-use was imprecise and included the null (OR 1.09, 95% CI 0.88–1.36; p = 0.44; I² = 68.1%; six estimates from five studies) [19,20,23,26,27]. Dual use was associated with higher odds of prevalent hypertension versus never or non-use (OR 1.41, 95% CI 1.16–1.72; p = 0.0005; I² = 80.9%; seven estimates from six studies) [[18], [19], [20],^23^,^26^,^27^].

**Fig. 2:**
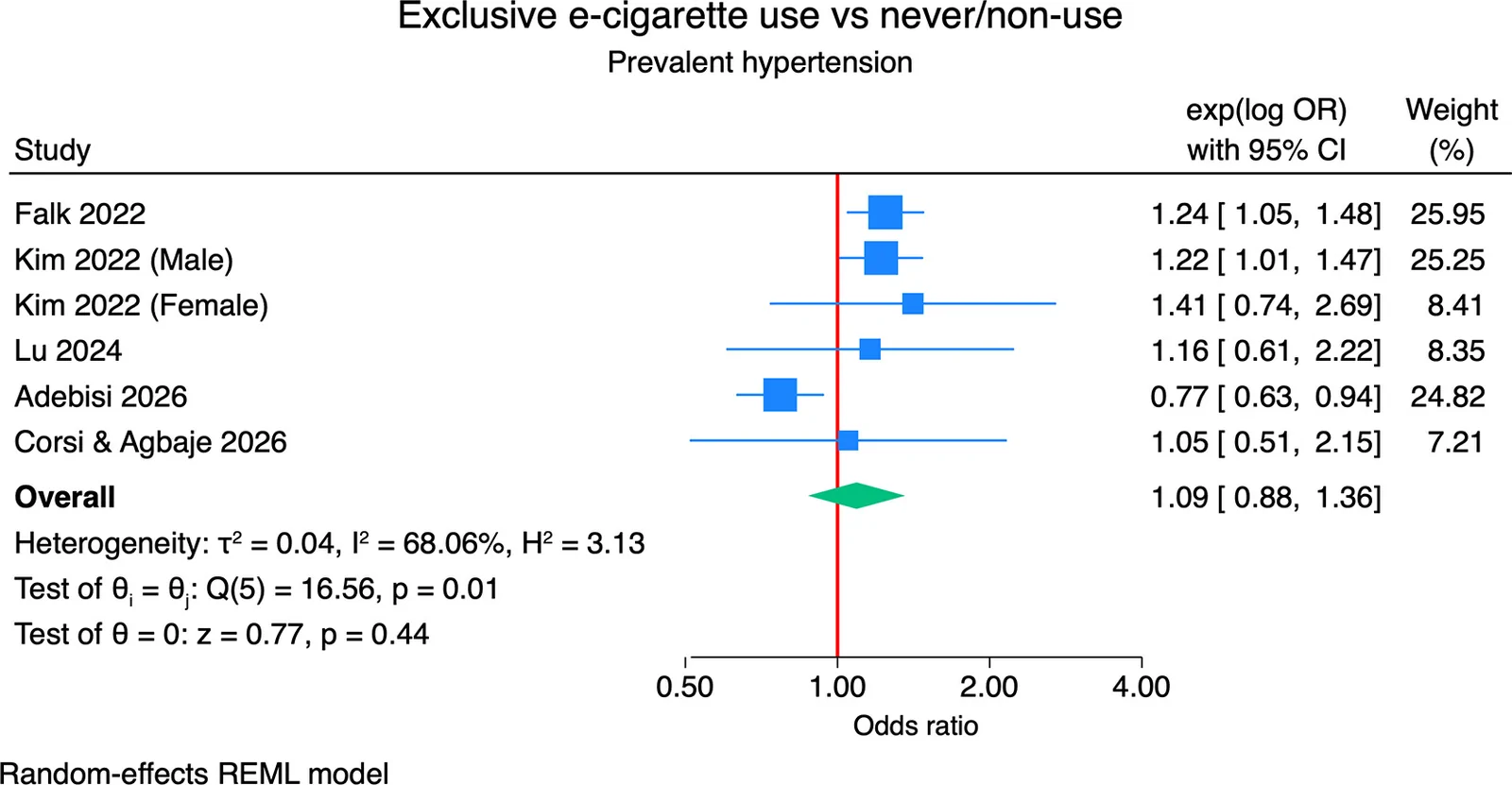
Forest plot of exclusive e-cigarette use versus never/non-use for prevalent hypertension.

**Fig. 3:**
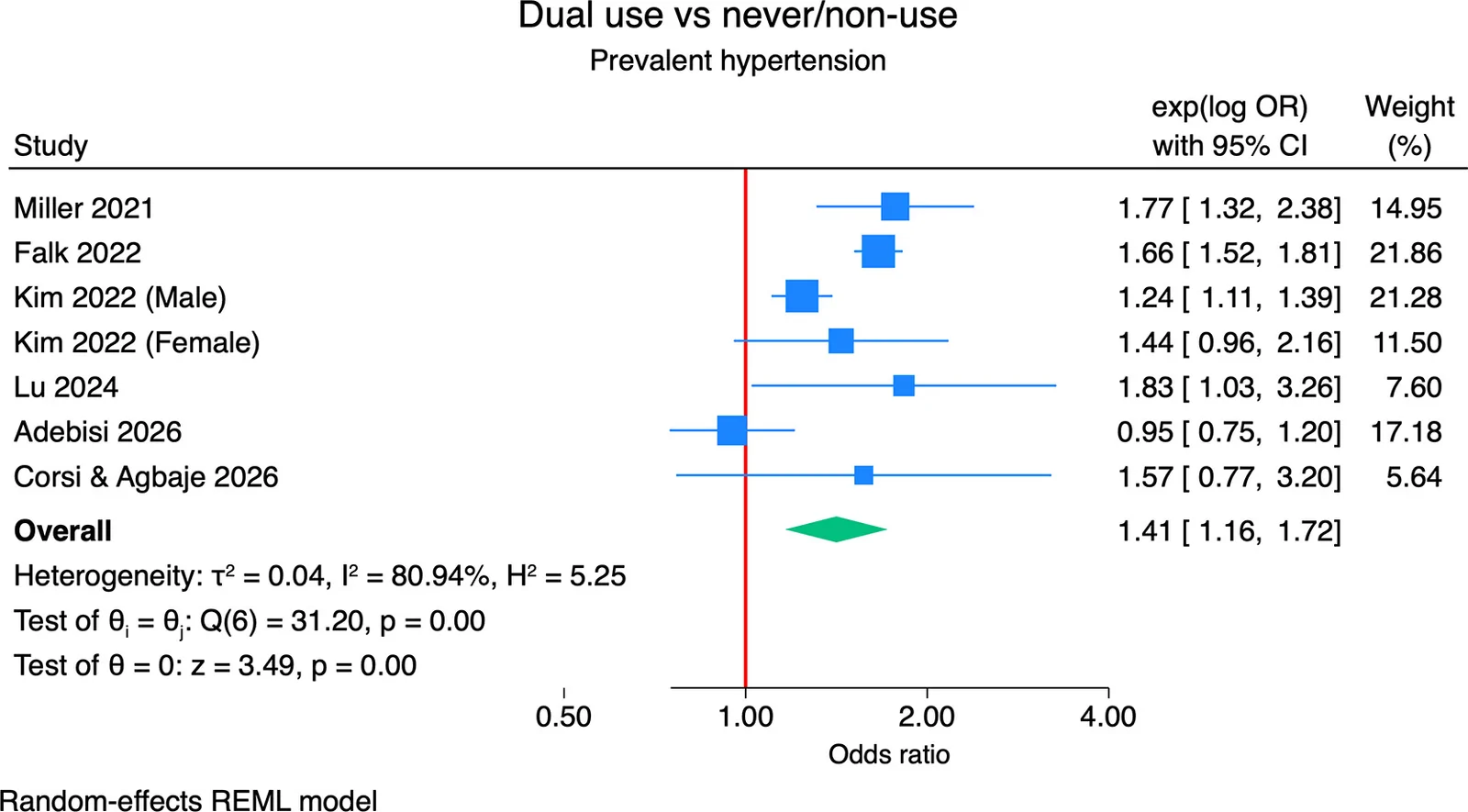
Forest plot of dual use versus never/non-use for prevalent hypertension.

**Fig. 4:**
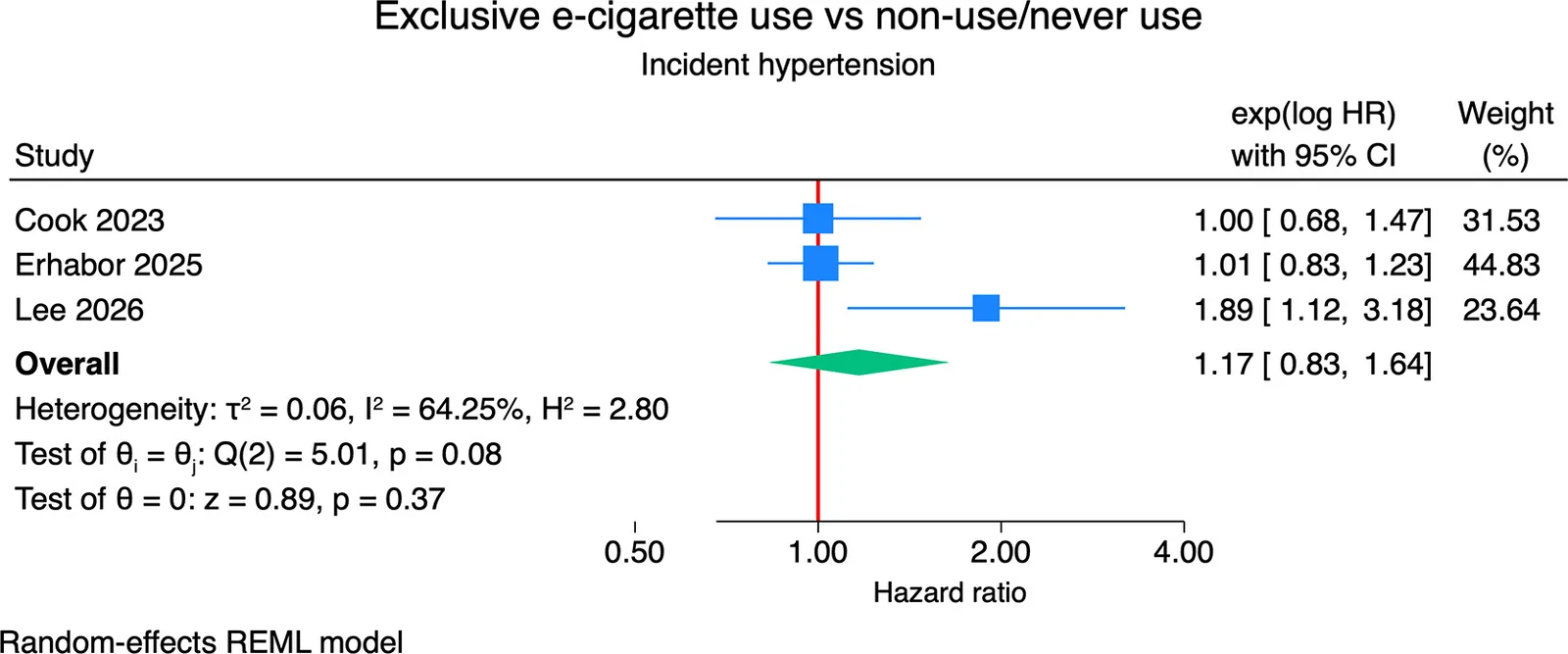
Forest plot of exclusive e-cigarette use versus non-use/never e-cigarette use for incident hypertension.

**Fig. 5:**
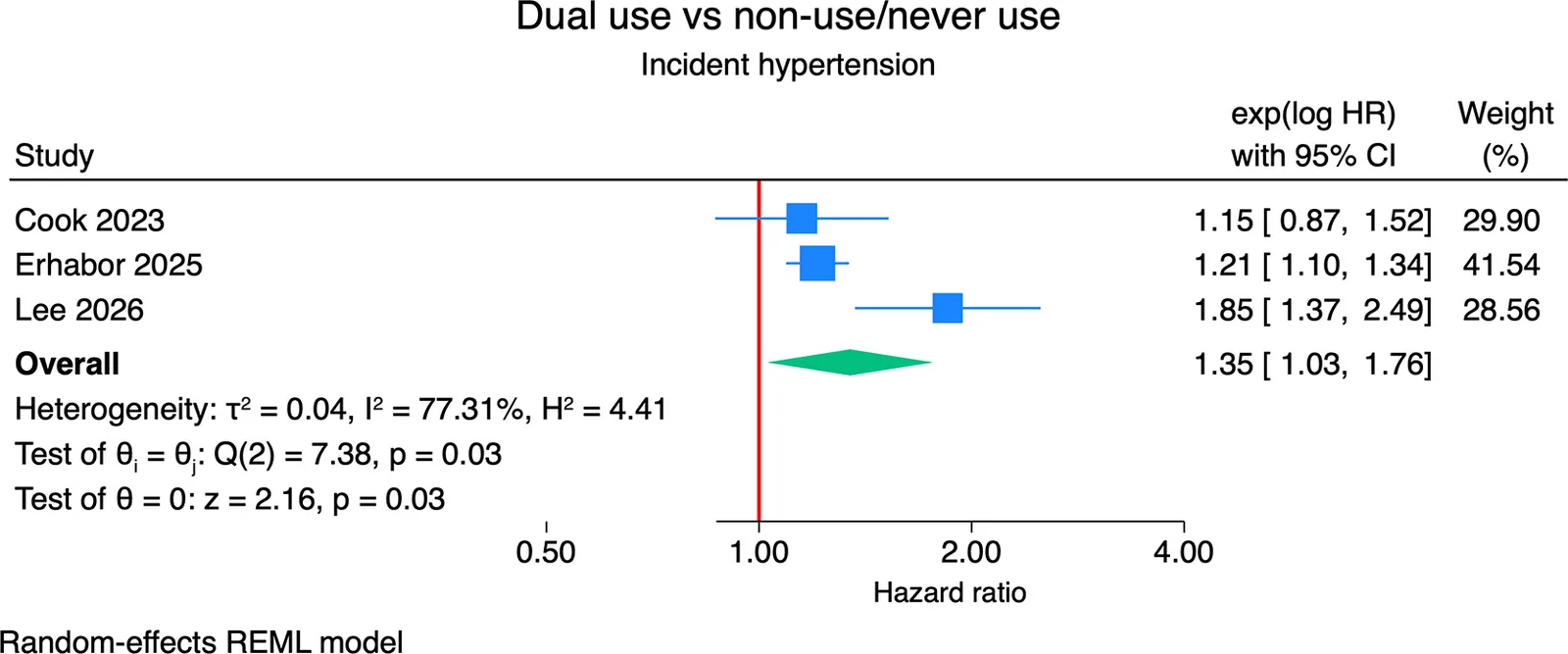
Forest plot of dual use versus non-use/never use for incident hypertension.

For incident hypertension, the pooled estimate for exclusive e-cigarette use versus non-use or never e-cigarette use was also imprecise and included the null (HR 1.17, 95% CI 0.83–1.64; p = 0.37; I² = 64.3%; three estimates from three studies) [22,24,25]. Dual use was associated with higher incident hypertension risk versus non-use or never use (HR 1.35, 95% CI 1.03–1.76; p = 0.031; I² = 77.3%; three estimates from three studies) [22,24,25]. Heterogeneity was moderate to substantial across all four comparisons.

### 3.4 Secondary and sensitivity analyses

Full secondary and sensitivity results are provided in Supplementary Figures S1–S7. In secondary comparator analyses, cigarette-only smoking versus never or non-use was associated with higher odds of prevalent hypertension (OR 1.25, 95% CI 1.10–1.41; I² = 87.8%) and higher risk of incident hypertension (HR 1.20, 95% CI 1.15–1.25; I² = 0.01%), with the incident result consistent across the two contributing cohorts. The former-smoker/current-vaper subgroup versus never or non-use showed no clear pooled association with prevalent hypertension and substantial inconsistency (OR 1.11, 95% CI 0.76–1.63; I² = 89.9%). Because cigarette-only and dual-use estimates came from separate comparisons rather than direct head-to-head contrasts, their pooled estimates cannot quantify whether adding e-cigarettes to cigarette smoking contributed additional hypertension risk.

In sensitivity analyses restricted to diagnosed or self-reported prevalent hypertension, exclusive e-cigarette use versus never/non-use remained non-significant with very high heterogeneity (OR 0.98, 95% CI 0.61–1.57; I² = 92.4%), and dual use versus never/non-use showed a positive but non-significant association (OR 1.41, 95% CI 0.96–2.06; I² = 90.7%).

Additional sensitivity analyses addressed overlap between longitudinal PATH studies by replacing the primary PATH estimate with sex-stratified PATH estimates from Shi et al. For incident hypertension, replacing Cook et al. with Shi et al. did not materially change the findings: exclusive e-cigarette use versus non-use/never use remained non-significant (HR 1.27, 95% CI 0.93–1.73; I² = 53.6%), while dual use versus non-use/never use remained associated with higher incident hypertension risk (HR 1.37, 95% CI 1.08–1.76; I² = 65.7%). These analyses are shown in Supplementary Figures S6 and S7.

### 3.5 Risk of bias

Study-level ROBINS-E judgements are presented in Supplementary Table S5. Most cross-sectional analyses were judged to be at high risk of bias, driven principally by reverse temporality because exposure and outcome were measured simultaneously, residual confounding, particularly by smoking history and adiposity, and exposure or outcome misclassification [[18], [19], [20],^23^,^26^,^27^]. Longitudinal studies were generally judged to raise some concerns rather than to be at low risk, because of residual confounding, self-reported exposure or outcome ascertainment, comparatively short follow-up for a chronic outcome, or selected study populations [21,22,24,25]. No study was judged to be at low overall risk of bias.

### 3.6 Certainty of evidence

GRADE certainty for the primary comparisons is summarised in Table 2, with full domain-level judgements presented in Supplementary Table S6. Certainty was very low for all four primary comparisons. Downgrading reflected risk of bias, inconsistency due to substantial heterogeneity, indirectness arising from differences in exposure and outcome definitions, and imprecision where confidence intervals included or approached the null.

**Table 2:** GRADE certainty of evidence for the primary comparisons.

Comparison	Pooled effect (95% CI)	Main reasons for downgrading	Certainty
Exclusive e-cigarette use vs never/non-use, prevalent hypertension	OR 1.09 (0.88–1.36)	Serious risk of bias; serious inconsistency (I² = 68.1%); indirectness from differing outcome definitions; serious imprecision (CI includes null)	Very low
Dual use vs never/non-use, prevalent hypertension	OR 1.41 (1.16–1.72)	Serious risk of bias; substantial inconsistency (I² = 80.9%); indirectness; CI excludes null	Very low
Exclusive e-cigarette use vs non-use/never use, incident hypertension	HR 1.17 (0.83–1.64)	Few studies; serious inconsistency (I² = 64.3%); imprecision (CI includes null)	Very low
Dual use vs non-use/never use, incident hypertension	HR 1.35 (1.03–1.76)	Few studies; serious inconsistency (I² = 77.3%); residual confounding; lower CI close to null	Very low

GRADE Grading of Recommendations Assessment, Development and Evaluation. Publication bias was not formally assessed because each pooled analysis included fewer than ten estimates.

## 4 Discussion

This systematic review included ten population-based observational studies. Exclusive e-cigarette use was not significantly associated with prevalent or incident hypertension. However, the estimates were imprecise, and the confidence intervals did not exclude potentially important increases in risk. Dual use was associated with higher odds of prevalent hypertension and a higher risk of incident hypertension, while cigarette-only smoking showed positive associations in secondary comparator analyses. These findings distinguish exposure patterns but do not establish causality. The non-significant findings for exclusive e-cigarette use should not be interpreted as evidence of no cardiovascular risk or as proof that e-cigarettes are safe.

Previous reviews have mainly examined acute blood-pressure responses, vascular-function markers, or broad cardiovascular outcomes [5,10,[28], [29], [30], [31]]. The present review addresses a narrower epidemiological question by treating hypertension as a defined population-based outcome, separating prevalent from incident disease, distinguishing exclusive e-cigarette use from dual use, and avoiding pooling across incompatible effect measures or overlapping cohorts. This structure is important because acute haemodynamic changes, cross-sectional hypertension prevalence, and new hypertension during follow-up are related but methodologically distinct outcomes.

Interpretation of exclusive e-cigarette use is constrained by prior smoking. Many exclusive users are former smokers and may retain risk associated with cigarettes smoked per day, years of smoking, age at initiation, cumulative pack-years, and time since cessation. These dimensions were measured inconsistently and were absent from several adjustment models. Differences in cessation motivation, body weight, baseline health, and healthcare contact may also produce residual confounding, healthy-switcher bias, or reverse causation. Consequently, the pooled estimates cannot isolate an effect of e-cigarette use that is independent of previous combustible-cigarette exposure [32].

Heterogeneity was moderate to substantial. The most evident between-study differences concerned exposure definitions and windows, inclusion of former smokers within exclusive-use groups, hypertension ascertainment, population age and country, and covariate adjustment. Self-reported diagnosis depends on awareness, healthcare access, and recall, whereas measured thresholds are influenced by measurement protocols, medication exclusions, and short-term variability; composite outcomes identify a broader case group. With only three to seven estimates per primary comparison, meta-regression or multiple subgroup analyses would have been unreliable, so the contribution of any single source of heterogeneity could not be quantified.

The inverse association in the Scottish pooled cross-sectional study contributed to heterogeneity in the prevalent exclusive-use analysis [26], but should not be interpreted as a protective biological effect. Its direction may reflect switching patterns, selection, diagnosis, or residual smoking-history confounding. The inconsistent former-smoker/current-vaper analysis further shows why future studies should distinguish never-smoking from former-smoking exclusive e-cigarette users.

The dual-use association is biologically plausible because dual users retain exposure to combustion-derived toxicants while often sustaining nicotine exposure. Nicotine-containing aerosols can activate sympathetic pathways and alter autonomic control, producing acute increases in heart rate and blood pressure. Repeated exposure may also contribute to oxidative stress, inflammatory signalling, endothelial dysfunction, arterial stiffness, and vascular remodelling; non-nicotine aerosol constituents may participate in some of these pathways, and effects may vary by device and liquid characteristics [[4], [5], [6],^33^]. Nevertheless, dual users differ from non-users in smoking intensity, nicotine dependence, alcohol use, diet, socioeconomic position, comorbidity, and healthcare contact [34], so biological plausibility does not remove the possibility of confounding.

Longitudinal analyses better establish temporality than cross-sectional analyses, but the incident evidence remains limited. Three non-overlapping cohorts contributed to each primary incident comparison, with follow-up of approximately 3.7–6.0 years and non-uniform exposure definitions. Replacing the primary PATH estimate with sex-stratified estimates from the overlapping PATH study did not materially alter the pattern: the exclusive-use estimate continued to include the null, whereas the dual-use estimate remained positive. Longer follow-up is still needed because hypertension may develop after prolonged exposure and product-use patterns can change over time.

Cigarette-only smoking provided an internal secondary comparator and was associated with prevalent and incident hypertension. However, the cigarette-only and dual-use pooled effects were derived from different study-specific contrasts. Their numerical similarity or difference cannot be interpreted as the effect of adding e-cigarettes to smoking, and no indirect subtraction would provide a valid causal estimate. The comparator results instead show that associations were clearest in exposure categories that retained combustible-cigarette use, while remaining subject to the known complexities of smoking, cessation, body weight, selection, and health-behaviour confounding [[35], [36], [37]].

The most defensible interpretation is therefore cautious. Current evidence is insufficient to determine whether exclusive e-cigarette use increases, decreases, or has no effect on long-term hypertension risk. Dual use was associated with hypertension, but the certainty of this evidence is very low and the association should not be presented as causal. The findings support separating exclusive use from dual use in research and practice and avoiding reassurance based solely on statistical non-significance.

### 4.1 Strengths and limitations

This review has several strengths. Separate meta-analyses for prevalent and incident hypertension reduced methodological bias from combining cross-sectional and longitudinal outcomes and improved clinical interpretation of prevalence and risk. The review also distinguished exclusive e-cigarette use from dual use, treated cigarette-only smoking as a secondary comparator, used author-reported adjusted estimates, and avoided double-counting overlapping PATH cohorts. Sensitivity analyses used alternative sex-stratified PATH estimates, and odds ratios, hazard ratios, and prevalence ratios were not inappropriately combined. Risk of bias and certainty were assessed using ROBINS-E and GRADE.

The review also has limitations. The protocol was not prospectively registered. Most prevalent-hypertension evidence was cross-sectional and therefore vulnerable to reverse causation. Exposure definitions varied across current, recent, ever, former, exclusive, and dual use, and previous smoking intensity, duration, age at initiation, and time since cessation were incompletely reported. Hypertension ascertainment differed across self-report, measured blood pressure, medication use, EHR diagnosis, and composite definitions. The search covered four major biomedical and multidisciplinary databases plus citation tracking but did not include every specialist database; studies indexed exclusively elsewhere may have been missed. Each pooled analysis contained few estimates, precluding reliable assessment of publication bias and limiting formal exploration of heterogeneity. Information on e-cigarette duration, frequency, nicotine concentration, device type, and switching history was often unavailable. Finally, restricting eligibility to dichotomous hypertension outcomes reduced comparability with studies of continuous blood-pressure change and may have omitted informative evidence on subthreshold physiological effects.

### 4.2 Implications for practice, policy, and research

The very low-certainty evidence does not justify a new clinical monitoring recommendation beyond established cardiovascular care, nor does it support reassurance that e-cigarette use is risk-free. Clinicians should ascertain cigarette and e-cigarette use and measure blood pressure as part of routine guideline-based cardiovascular risk assessment, with particular attention to people who continue dual use. For people who smoke and use e-cigarettes, the priority remains complete discontinuation of combustible cigarettes rather than sustained dual use.

Future studies should use standardised exposure and outcome definitions and should separate exclusive e-cigarette users who never smoked from those who formerly smoked. Prior cigarette exposure should be characterised by cigarettes per day, duration, age at initiation, pack-years, time since cessation, and switching pathway. E-cigarette frequency, duration, device type, liquid characteristics, and nicotine exposure should also be recorded. Hypertension ascertainment should use validated diagnoses, repeated measured blood pressure, medication data, or linked EHR records. Longer prospective studies with repeated exposure assessment are needed to capture changes in product use and the development of a chronic outcome.

## 5 Conclusion

Current population-based evidence did not show a statistically significant association between exclusive e-cigarette use and prevalent or incident hypertension. However, the certainty of evidence was very low, and the confidence intervals did not exclude potentially important increases in risk. These findings should therefore not be interpreted as proof that e-cigarettes carry no cardiovascular risk or are safe. Dual use of e-cigarettes and combustible cigarettes was associated with higher odds of prevalent hypertension and a higher risk of incident hypertension, although these observational associations were heterogeneous and vulnerable to residual confounding and exposure misclassification. Longer prospective studies using standardised exposure definitions, detailed prior smoking histories, repeated exposure assessment, and validated hypertension outcomes are needed.

## Funding

This work was funded by a grant from the Ministero dell’Università e della Ricerca (MUR), Bando PNRR 341/2022 (Heal Italy; CUP E63C22002080006), and was also supported by the Department of Clinical and Experimental Medicine, University of Catania (Departmental Fund 6C725202094). Yusuff Adebayo Adebisi is supported by a UKRI Economic and Social Research Council (ESRC) studentship [ES/P000681/1].

## Ethical approval

Not applicable. This is a systematic review and meta-analysis.

## Contributors

YAA conceived and designed the study, coordinated the review process, conducted the statistical analyses, and drafted the initial manuscript. YAA, HEN, DTA, and EI contributed to study screening, data extraction, and verification of extracted data. MV, JG, AKS, NZA, DC, BAA, DAB, AAH, and RP contributed to interpretation of the findings and critically revised the manuscript for important intellectual content. All authors reviewed and approved the final manuscript and agreed with the decision to submit it for publication.

## Data availability

This study is a systematic review and meta-analysis of previously published data. All data analysed were extracted from the included primary studies, which are cited in the manuscript and listed in the references. No new datasets were generated. The extracted study-level data supporting the findings, including the full extraction tables and estimates that were not pooled, are provided in the Supplementary Material (Tables S2 and S3). Additional data are available from the corresponding author on reasonable request.

## CRediT authorship contribution statement

**Yusuff Adebayo Adebisi:** Writing – review & editing, Writing – original draft, Visualization, Software, Project administration, Methodology, Investigation, Funding acquisition, Formal analysis, Data curation, Conceptualization. **Massimo Volpe:** Writing – review & editing, Writing – original draft, Methodology, Investigation. **Jacob George:** Writing – review & editing, Writing – original draft, Validation, Methodology, Investigation. **Ahmed K. Shukri:** Writing – review & editing, Writing – original draft, Resources, Methodology, Investigation. **Najim Z. Alshahrani:** Writing – review & editing, Writing – original draft, Visualization, Methodology, Investigation. **Davide Capodanno:** Writing – review & editing, Writing – original draft, Methodology, Investigation. **Bader Ali Almustafa:** Writing – review & editing, Writing – original draft, Visualization, Methodology, Investigation. **Anas Ali Alhur:** Writing – review & editing, Project administration, Methodology, Investigation. **Henry Eziefule Nwankwo:** Writing – review & editing, Writing – original draft, Visualization, Validation, Supervision. **Deborah Temitope Adeyemo:** Writing – review & editing, Writing – original draft, Visualization, Validation, Methodology, Investigation. **Eseosa Iyagbaye:** Writing – original draft, Visualization, Validation, Methodology, Investigation. **Duaa Abdullah Bafail:** Writing – review & editing, Writing – original draft, Visualization, Validation. **Riccardo Polosa:** Writing – review & editing, Writing – original draft, Visualization, Validation, Supervision, Software, Project administration, Methodology, Investigation, Data curation.

## Declaration of competing interest

Yusuff Adebayo Adebisi previously received funding through the Tobacco Harm Reduction Scholarship and the Kevin Molloy Fellowship, both awarded by Knowledge-Action-Change (KAC), UK. KAC has received funding from Global Action to End Smoking (GA), a U.S. nonprofit 501(c)(3) grant-making organisation that supports health and science research, cessation education, and agricultural transformation initiatives. Massimo Volpe has received honoraria for speakers’ bureaus or advisory boards from AstraZeneca, Menarini, GSK, Pfizer, Sanofi, and Servier, and is supported by a grant from the Italian Ministry of Health (“Ricerca Corrente”). Riccardo Polosa is a tenured Professor of Internal Medicine at the University of Catania, Italy, and Medical Director of the Institute for Internal Medicine at the same university. He has received EU and governmental competitive grants, including U-BIOPRED, AIR-PROM, Integral Rheumatology & Immunology Specialists Network (IRIS), and several PNRR grants funded by NextGenerationEU of the European Commission. He has also received investigator-initiated grants from GA, Pfizer, GlaxoSmithKline, CV Therapeutics, NeuroSearch A/S, Sandoz, Merck Sharp & Dohme, Boehringer Ingelheim, Novartis, Arbi Group Srl, Duska Therapeutics, and Forest Laboratories. He is the founder of the Center for Tobacco Prevention and Treatment (CPCT) and the Center of Excellence for the Acceleration of Harm Reduction at the University of Catania. He has received consultancy fees from Pfizer, Boehringer Ingelheim, Duska Therapeutics, Forest Laboratories, CV Therapeutics, Sermo Inc., GRG Health, Clarivate Analytics, Guidepoint Expert Network, and GLG Group. He receives textbook royalties from Elsevier and EDRA. He is Chair of the European Technical Committee for Standardisation on “Requirements and test methods for emissions of electronic cigarettes” (CEN/TC 437; WG4) and scientific adviser to the non-profit Foundation RIDE2Med. All other authors declare no competing interests.
