# Supplementary material for: Associations of exclusive e-cigarette use and dual use of e-cigarettes and combustible cigarettes with prevalent and incident hypertension: A systematic review and meta-analysis
Source: Am J Prev Cardiol. 2026 Jul 24;30:101767. doi: 10.1016/j.ajpc.2026.101767 (PMC13632134; doi:10.1016/j.ajpc.2026.101767)
Supplement: Supplementary file 1 [file mmc1.docx]

**Supplementary File**

*Associations of exclusive e-cigarette use and dual use of e-cigarettes and combustible cigarettes with prevalent and incident hypertension: A systematic review and meta-analysis*

**Table S1. Database search strategy**

| **Database** | **Search concept** | **Final search string and date** |
| --- | --- | --- |
| PubMed/MEDLINE | E-cigarette/vaping/ENDS terms AND hypertension/high-blood-pressure terms | Search conducted on **31 May 2026**. Final search string: (("electronic nicotine delivery systems"[MeSH Terms] OR "vaping"[MeSH Terms] OR "electronic cigarette*"[Title/Abstract] OR e-cigarette*[Title/Abstract] OR ecigarette*[Title/Abstract] OR vape[Title/Abstract] OR vapes[Title/Abstract] OR vaping[Title/Abstract] OR vaper*[Title/Abstract] OR ENDS[Title/Abstract] OR "electronic nicotine delivery system*"[Title/Abstract] OR "electronic nicotine device*"[Title/Abstract]) AND ("hypertension"[MeSH Terms] OR hypertension[Title/Abstract] OR hypertensive[Title/Abstract] OR "high blood pressure"[Title/Abstract] OR "raised blood pressure"[Title/Abstract] OR "blood pressure"[Title/Abstract])) |
| Embase | E-cigarette/vaping/ENDS terms AND hypertension/high-blood-pressure terms using Emtree and free-text terms | Search conducted on **31 May 2026**. Final search string: (exp electronic cigarette/ or exp vaping/ or (electronic cigarette* or e-cigarette* or ecigarette* or vape or vapes or vaping or vaper* or ENDS or electronic nicotine delivery system* or electronic nicotine device*).ti,ab,kw.) and (exp hypertension/ or exp blood pressure/ or (hypertension or hypertensive or high blood pressure or raised blood pressure or blood pressure).ti,ab,kw.) |
| Scopus | Title, abstract, and keyword search for e-cigarette/vaping/ENDS terms AND hypertension/high-blood-pressure terms | Search conducted on **31 May 2026**. Final search string: TITLE-ABS-KEY ( ( "electronic cigarette*" OR "e-cigarette*" OR "e cigarette*" OR ecigarette* OR vaping OR vaper* OR vape* OR "electronic nicotine delivery system*" OR "electronic nicotine device*" ) AND ( hypertension OR hypertensive OR "high blood pressure" OR "raised blood pressure" OR "systolic blood pressure" OR "diastolic blood pressure" ) ) |
| Web of Science Core Collection | Topic search for e-cigarette/vaping/ENDS terms AND hypertension/high-blood-pressure terms | Search conducted on **31 May 2026**. Final search string: TS=( ("electronic cigarette*" OR "e-cigarette*" OR "e cigarette*" OR ecigarette* OR vaping OR vaper* OR vape* OR "electronic nicotine delivery system*" OR "electronic nicotine device*") AND (hypertension OR hypertensive OR "high blood pressure" OR "raised blood pressure" OR "systolic blood pressure" OR "diastolic blood pressure") ) |
| Reference lists and citation tracking | Backward and forward citation screening of included studies and relevant systematic reviews | Conducted on **31 May 2026**. Reference lists of all included studies and relevant reviews were screened manually. Forward citation tracking was used to identify additional eligible population-based observational studies reporting exposure-specific estimates for e-cigarette use, dual use, cigarette-only smoking, and hypertension. Conference abstracts without a corresponding full-text peer-reviewed article were not included in the main synthesis. |

**Abbreviations:** ENDS, electronic nicotine delivery systems; MeSH, Medical Subject Headings; Emtree, Embase subject headings; ti, title; ab, abstract; kw, keyword. No restrictions were applied by publication year. Studies were eligible for inclusion if they reported extractable exposure-specific estimates for hypertension defined by self-report, doctor or health-professional diagnosis, electronic health record diagnosis, antihypertensive medication use, or measured blood pressure thresholds. Acute blood-pressure experiments, continuous blood-pressure-change studies, metabolic-syndrome blood-pressure components, and studies without extractable exposure-specific hypertension estimates were excluded. The protocol was not prospectively registered.

**Table S2.** Detailed characteristics of included studies

| **Study** | **Country / data source** | **Design / period** | **Population / sample** | **Exposure definition** | **Hypertension outcome** | **Adjustment** | **Review-use note** |
| --- | --- | --- | --- | --- | --- | --- | --- |
| Miller et al., 2021  <https://www.mdpi.com/2305-6304/9/3/52> | USA; PATH Study Wave 3 | Cross-sectional; 2015–2016 | Adults 18–54 y; excluded ≥55 y and established users of other tobacco products; n=19,147 | Current vaping; current smoking; six-category status (exclusive vaper never/former smoker, dual user, exclusive smoker, former smoker, never smoker) | Self-reported diagnosed hypertension/high blood pressure (current or controlled) | Survey-weighted logistic regression: age, sex, race/ethnicity, education, income, insurance, marital status, physical activity, BMI, heavy alcohol use, hypercholesterolaemia, diabetes | Prevalent self-reported hypertension; potential residual smoking-history and switcher bias |
| Falk et al., 2022  <https://journals.ku.edu/kjm/article/view/16752> | USA; NHIS | Cross-sectional; 2014, 2016, 2017, 2018 | Adults ≥18 y completing the tobacco questionnaire; n=84,553 | E-cigarette only; cigarette only; former smoker currently vaping; former smoker not vaping; dual use | Self-reported physician/health-professional diagnosis of hypertension/high blood pressure | Sex, BMI, age | Prevalent self-reported hypertension; limited adjustment set |
| Kim et al., 2022  <https://www.frontiersin.org/journals/public-health/articles/10.3389/fpubh.2022.919585/full> | S. Korea; Korea Community Health Survey | Cross-sectional; 2019 | Adults ≥19 y; antihypertensive-medication users excluded; n=275,762 | Dual smoker; e-cigarette only; conventional cigarette only; past smoker; non-smoker | Measured: SBP ≥140 or DBP ≥90 mmHg (mean of three readings) | Sex-stratified logistic regression: demographic, socioeconomic, behavioural, and health factors | Measured hypertension; keep sex-specific estimates separate |
| Shi et al., 2023  <https://academic.oup.com/ntr/article/25/3/478/6648174> | USA; PATH Study Waves 1–4 | Prospective cohort; 2013/14 baseline, follow-up to 2018 | Adults ≥18 y, no lifetime hypertension at baseline, complete Waves 2–4; n=16,434 | Time-varying, time-lagged exclusive e-cigarette, exclusive cigarette, dual, and non-use; dose-response (intermittent/consistent) | Self-reported incident diagnosis in past 12 months | Sex-stratified weighted Cox: education, other tobacco use, BMI, physical activity, heavy drinking, diabetes, hypercholesterolaemia, CVD, baseline age, race/ethnicity, lifetime cigarette exposure, family history | Incident; overlaps PATH with Cook; do not double-count |
| Cook et al., 2023  <https://bmjopen.bmj.com/content/13/4/e062297> | USA; PATH Study Waves 1–5 | Prospective longitudinal cohort; 2013–2019 | Adults ≥18 y, no baseline heart condition, hypertension, or high cholesterol; n=17,539 | Time-varying, one-wave-lagged non-use, exclusive cigarette, exclusive ENDS, dual use | Self-reported incident hypertension diagnosis | Discrete-time survival: age, sex, race/ethnicity, income, family history of heart attack, obesity, diabetes, binge drinking, former established smoking, pack-years | Primary longitudinal PATH estimate |
| Lu et al., 2024  <https://www.tobaccoinduceddiseases.org/Association-of-the-use-of-e-cigarettes-combustible-cigarettes-or-dual-use-with-hypertension,195397,0,2.html> | USA; NHANES | Cross-sectional with mortality follow-up; 2015–2018 | Adults ≥20 y with complete data; n=7,696 | Never/former/current e-cigarette and combustible cigarette use; dual-use categories | Measured SBP ≥140 or DBP ≥90 mmHg, self-report, or antihypertensive medication | Model 3: age, sex, race, marital status, education, poverty-to-income ratio, BMI, alcohol, coronary heart disease, diabetes | Prevalent composite hypertension; subgroup by outcome definition recommended |
| Erhabor et al., 2025  <https://academic.oup.com/ntr/article-abstract/27/9/1651/8079085> | USA; All of Us Research Program | Longitudinal, EHR-linked; median follow-up 3.7–3.9 y | Adults with linked EHR data; former e-cigarette/cigarette users excluded; n=249,190 | Non-use; exclusive e-cigarette; exclusive combustible cigarette; dual use | Incident EHR-recorded hypertension (standardised medical terms) | Cox regression: age, sex, race/ethnicity, BMI | Incident EHR-recorded hypertension; large cohort; limited adjustment |
| Lee et al., 2026  <https://www.jacc.org/doi/10.1016/j.jacadv.2025.102525> | USA; HCHS/SOL | Population-based cohort; Visit 2 2014–2017, Visit 3 2020–2024; median follow-up 6.0 y | Hispanic/Latino adults 18–74 y; n=11,593 prevalent, 6,306 incident | Ever/former/current e-cigarette use; combined e-cigarette and combustible cigarette use | Prevalent and incident: measured BP ≥140/90 mmHg or antihypertensive treatment | Model 2: baseline BP (incident), age, sex, Hispanic/Latino background, combustible smoking, alcohol, education, income, BMI, diabetes, total and HDL cholesterol | Prevalent and incident clinical hypertension; Hispanic/Latino population |
| Adebisi et al., 2026  <https://www.nature.com/articles/s41371-026-01171-4> | Scotland; Scottish Health Survey | Pooled cross-sectional; 2017, 2018, 2019, 2021, 2022 | Adults ≥16 y in private households; n=22,187 | Never users; former smokers; cigarette only; e-cigarette only; dual use; e-cig-only split by smoking history | Self-reported doctor-diagnosed high blood pressure (excluding pregnancy) | Age group, sex, survey year, area deprivation, education, ethnicity, diabetes, other CVD, alcohol, physical activity | Prevalent doctor-diagnosed hypertension; inverse association attenuated in PS-matched sensitivity analyses |
| Corsi & Agbaje, 2026  <https://journals.physiology.org/doi/full/10.1152/ajpheart.00958.2025> | USA; NHANES | Cross-sectional; 2021–2023 | Participants ≥12 y with complete smoking/vaping status; n=6,262 | Any smoking/vaping; exclusive smoker; exclusive vaper; dual user (recent 5-day exposure) | Measured: SBP ≥140 or DBP ≥90 mmHg | Age, sex, education, race/ethnicity, BMI, total cholesterol, plasma glucose, C-reactive protein, physical activity | Measured hypertension; analytically distinct (5-day exposure window, includes adolescents) |

*PATH Population Assessment of Tobacco and Health; NHIS National Health Interview Survey; NHANES National Health and Nutrition Examination Survey; HCHS/SOL Hispanic Community Health Study/Study of Latinos; ENDS electronic nicotine delivery systems; EHR electronic health record; BMI body-mass index; CVD cardiovascular disease; HDL high-density lipoprotein; SBP systolic blood pressure; DBP diastolic blood pressure; PS propensity score.*

**Table S3.** Full extraction of author-reported effect estimates for hypertension

*All estimates are reported as published by the original studies; no estimates were recalculated by the reviewers. Comparisons labelled secondary, subgroup, sensitivity, or dose-response were not pooled with the primary estimates unless pre-specified.*

| **Study** | **Outcome type** | **Exposure group** | **Comparator** | **Measure** | **Estimate** | **95% CI** | **Adjustment / model** |
| --- | --- | --- | --- | --- | --- | --- | --- |
| Miller 2021 | Self-reported diagnosed HTN | Current vaper | Not current vaper | aOR | 1.31 | 1.05–1.63 | Survey-weighted multivariable logistic regression (Table S2) |
| Miller 2021 | Self-reported diagnosed HTN | Current smoker | Not current smoker | aOR | 1.27 | 1.10–1.47 | Survey-weighted multivariable logistic regression (Table S2) |
| Miller 2021 | Self-reported diagnosed HTN | Exclusive vaper, never smoker | Never smoker | aOR | 1.32 | 0.50–3.53 | Survey-weighted multivariable logistic regression (Table S2) |
| Miller 2021 | Self-reported diagnosed HTN | Exclusive vaper, former smoker | Never smoker | aOR | 1.42 | 0.98–2.06 | Survey-weighted multivariable logistic regression (Table S2) |
| Miller 2021 | Self-reported diagnosed HTN | Former smoker | Never smoker | aOR | 1.28 | 1.05–1.57 | Survey-weighted multivariable logistic regression (Table S2) |
| Miller 2021 | Self-reported diagnosed HTN | Exclusive smoker | Never smoker | aOR | 1.36 | 1.15–1.62 | Survey-weighted multivariable logistic regression (Table S2) |
| Miller 2021 | Self-reported diagnosed HTN | Dual user | Never smoker | aOR | 1.77 | 1.32–2.39 | Survey-weighted multivariable logistic regression (Table S2) |
| Miller 2021 | Self-reported diagnosed HTN | Exclusive vaper, never smoker | Former smoker | aOR | 1.03 | 0.38–2.83 | Survey-weighted multivariable logistic regression (Table S2) |
| Miller 2021 | Self-reported diagnosed HTN | Exclusive vaper, former smoker | Former smoker | aOR | 1.11 | 0.74–1.66 | Survey-weighted multivariable logistic regression (Table S2) |
| Miller 2021 | Self-reported diagnosed HTN | Exclusive smoker | Former smoker | aOR | 1.06 | 0.87–1.30 | Survey-weighted multivariable logistic regression (Table S2) |
| Miller 2021 | Self-reported diagnosed HTN | Dual user | Former smoker | aOR | 1.38 | 0.98–1.96 | Survey-weighted multivariable logistic regression (Table S2) |
| Miller 2021 | Self-reported diagnosed HTN | Exclusive vaper, never smoker | Exclusive smoker | aOR | 0.96 | 0.37–2.57 | Survey-weighted multivariable logistic regression (Table S2) |
| Miller 2021 | Self-reported diagnosed HTN | Exclusive vaper, former smoker | Exclusive smoker | aOR | 1.04 | 0.72–1.51 | Survey-weighted multivariable logistic regression (Table S2) |
| Miller 2021 | Self-reported diagnosed HTN | Dual user | Exclusive smoker | aOR | 1.30 | 0.99–1.71 | Survey-weighted multivariable logistic regression (Table S2) |
| Falk 2022 | Self-reported diagnosed HTN | E-cigarette only users | Never users (combustible or e-cig) | OR | 1.244 | 1.048–1.477 | Sex, BMI, age |
| Falk 2022 | Self-reported diagnosed HTN | Combustible cigarette only users | Never users | OR | 1.384 | 1.277–1.499 | Sex, BMI, age |
| Falk 2022 | Self-reported diagnosed HTN | Former smoker currently using e-cigarettes | Never users | OR | 1.308 | 1.162–1.472 | Sex, BMI, age |
| Falk 2022 | Self-reported diagnosed HTN | Former smoker not currently using e-cigarettes | Never users | OR | 1.139 | 1.082–1.198 | Sex, BMI, age |
| Falk 2022 | Self-reported diagnosed HTN | Dual users | Never users | OR | 1.660 | 1.519–1.814 | Sex, BMI, age |
| Kim 2022 | Measured HTN/elevated BP | Dual smoker, male | Male non-smoker | OR | 1.24 | 1.11–1.39 | Sex-stratified adjusted logistic regression |
| Kim 2022 | Measured HTN/elevated BP | E-cigarette only, male | Male non-smoker | OR | 1.22 | 1.02–1.48 | Sex-stratified adjusted logistic regression |
| Kim 2022 | Measured HTN/elevated BP | Conventional cigarette only, male | Male non-smoker | OR | 1.16 | 1.11–1.22 | Sex-stratified adjusted logistic regression |
| Kim 2022 | Measured HTN/elevated BP | Past smoker, male | Male non-smoker | OR | 1.09 | 1.04–1.14 | Sex-stratified adjusted logistic regression |
| Kim 2022 | Measured HTN/elevated BP | Dual smoker, female | Female non-smoker | OR | 1.44 | 0.96–2.16 | Sex-stratified adjusted logistic regression |
| Kim 2022 | Measured HTN/elevated BP | E-cigarette only, female | Female non-smoker | OR | 1.41 | 0.74–2.70 | Sex-stratified adjusted logistic regression |
| Kim 2022 | Measured HTN/elevated BP | Conventional cigarette only, female | Female non-smoker | OR | 1.35 | 1.24–1.48 | Sex-stratified adjusted logistic regression |
| Kim 2022 | Measured HTN/elevated BP | Past smoker, female | Female non-smoker | OR | 1.23 | 1.11–1.35 | Sex-stratified adjusted logistic regression |
| Shi 2023 | Incident self-reported HTN, male | Exclusive e-cigarette use | Non-use | aHR | 1.06 | 0.58–1.94 | Sex-stratified weighted Cox (Table S3) |
| Shi 2023 | Incident self-reported HTN, male | Exclusive cigarette use | Non-use | aHR | 1.12 | 0.84–1.49 | Sex-stratified weighted Cox |
| Shi 2023 | Incident self-reported HTN, male | Dual use | Non-use | aHR | 1.02 | 0.65–1.59 | Sex-stratified weighted Cox |
| Shi 2023 | Incident self-reported HTN, female | Exclusive e-cigarette use | Non-use | aHR | 1.54 | 0.96–2.48 | Sex-stratified weighted Cox |
| Shi 2023 | Incident self-reported HTN, female | Exclusive cigarette use | Non-use | aHR | 1.69 | 1.21–2.34 | Sex-stratified weighted Cox |
| Shi 2023 | Incident self-reported HTN, female | Dual use | Non-use | aHR | 1.56 | 1.04–2.34 | Sex-stratified weighted Cox |
| Shi 2023 | Incident self-reported HTN, male | Intermittent exclusive e-cig use | Consistent non-use | aHR | 0.89 | 0.48–1.64 | Sex-stratified weighted Cox |
| Shi 2023 | Incident self-reported HTN, male | Consistent exclusive e-cig use | Consistent non-use | aHR | 1.17 | 0.56–2.46 | Sex-stratified weighted Cox |
| Shi 2023 | Incident self-reported HTN, male | Intermittent dual use | Consistent non-use | aHR | 0.71 | 0.40–1.26 | Sex-stratified weighted Cox |
| Shi 2023 | Incident self-reported HTN, male | Consistent dual use | Consistent non-use | aHR | 1.18 | 0.65–2.14 | Sex-stratified weighted Cox |
| Shi 2023 | Incident self-reported HTN, female | Intermittent exclusive e-cig use | Consistent non-use | aHR | 0.89 | 0.43–1.83 | Sex-stratified weighted Cox |
| Shi 2023 | Incident self-reported HTN, female | Consistent exclusive e-cig use | Consistent non-use | aHR | 1.84 | 0.96–3.52 | Sex-stratified weighted Cox |
| Shi 2023 | Incident self-reported HTN, female | Intermittent dual use | Consistent non-use | aHR | 0.86 | 0.44–1.68 | Sex-stratified weighted Cox |
| Shi 2023 | Incident self-reported HTN, female | Consistent dual use | Consistent non-use | aHR | 1.71 | 1.00–2.93 | Sex-stratified weighted Cox |
| Cook 2023 | Self-reported incident HTN | Exclusive cigarette use | Non-use | aHR | 1.21 | 1.06–1.38 | Discrete-time survival (Table S2) |
| Cook 2023 | Self-reported incident HTN | Exclusive ENDS use | Non-use | aHR | 1.00 | 0.68–1.47 | Discrete-time survival |
| Cook 2023 | Self-reported incident HTN | Dual use | Non-use | aHR | 1.15 | 0.87–1.52 | Discrete-time survival |
| Lu 2024 | Prevalent HTN | Former e-cigarette use | Never e-cigarette use | AOR | 1.40 | 1.09–1.81 | Model 3 (Table S2) |
| Lu 2024 | Prevalent HTN | Current e-cigarette use | Never e-cigarette use | AOR | 1.56 | 1.01–2.42 | Model 3 |
| Lu 2024 | Prevalent HTN | Former combustible cigarette use | Never combustible cigarette use | AOR | 0.98 | 0.80–1.19 | Model 3 |
| Lu 2024 | Prevalent HTN | Current combustible cigarette use | Never combustible cigarette use | AOR | 1.29 | 1.01–1.64 | Model 3 |
| Lu 2024 | Prevalent HTN | Combustible cigarette only use | Never cigarette use | AOR | 1.25 | 0.91–1.73 | Model 3 |
| Lu 2024 | Prevalent HTN | E-cigarette only use | Never cigarette use | AOR | 1.16 | 0.61–2.24 | Model 3 |
| Lu 2024 | Prevalent HTN | Dual use | Never cigarette use | AOR | 1.83 | 1.03–3.27 | Model 3 |
| Erhabor 2025 | Incident HTN | Exclusive e-cigarette use | Non-use | aHR | 1.01 | 0.83–1.23 | Cox: age, sex, race/ethnicity, BMI |
| Erhabor 2025 | Incident HTN | Exclusive combustible cigarette use | Non-use | aHR | 1.20 | 1.15–1.26 | Cox: age, sex, race/ethnicity, BMI |
| Erhabor 2025 | Incident HTN | Dual use | Non-use | aHR | 1.21 | 1.10–1.34 | Cox: age, sex, race/ethnicity, BMI |
| Erhabor 2025 | Incident HTN, age-restricted | Exclusive e-cig use, 30–70 y | Non-use, 30–70 y | aHR | 1.39 | 1.09–1.77 | Cox: age, sex, race/ethnicity, BMI |
| Lee 2026 | Prevalent HTN | Ever e-cigarette use | Never e-cigarette use | aPR | 0.86 | 0.73–1.02 | Model 2 (Table S2) |
| Lee 2026 | Prevalent HTN | Former e-cigarette use | Never e-cigarette use | aPR | 0.83 | 0.69–1.00 | Model 2 |
| Lee 2026 | Prevalent HTN | Current e-cigarette use | Never e-cigarette use | aPR | 0.99 | 0.66–1.46 | Model 2 |
| Lee 2026 | Incident HTN | Ever e-cigarette use | Never e-cigarette use | aHR | 1.49 | 1.14–1.95 | Model 2 |
| Lee 2026 | Incident HTN | Former e-cigarette use | Never e-cigarette use | aHR | 1.43 | 1.07–1.93 | Model 2 |
| Lee 2026 | Incident HTN | Current e-cigarette use | Never e-cigarette use | aHR | 1.89 | 1.12–3.18 | Model 2 |
| Lee 2026 | Incident HTN | Dual ever use | Never users | aHR | 1.85 | 1.37–2.49 | Model 2 |
| Lee 2026 | Incident HTN, age subgroup | Ever e-cig use, <50 y | Never e-cig use, <50 y | aHR | 1.78 | 1.25–2.53 | Model 2 |
| Lee 2026 | Incident HTN, age subgroup | Ever e-cig use, ≥50 y | Never e-cig use, ≥50 y | aHR | 0.82 | 0.55–1.24 | Model 2 |
| Lee 2026 | Incident HTN, sex subgroup | Ever e-cig use, male | Never e-cig use, male | aHR | 1.69 | 1.15–2.49 | Model 2 |
| Lee 2026 | Incident HTN, sex subgroup | Ever e-cig use, female | Never e-cig use, female | aHR | 1.06 | 0.69–1.64 | Model 2 |
| Lee 2026 | Incident HTN, age subgroup | Current e-cig use, <50 y | Never/former e-cig use, <50 y | aHR | 2.16 | 1.10–4.24 | Model 2 |
| Lee 2026 | Incident HTN, age subgroup | Current e-cig use, ≥50 y | Never/former e-cig use, ≥50 y | aHR | 1.25 | 0.77–2.02 | Model 2 |
| Lee 2026 | Incident HTN, sex subgroup | Current e-cig use, male | Never/former e-cig use, male | aHR | 1.91 | 1.04–3.50 | Model 2 |
| Lee 2026 | Incident HTN, sex subgroup | Current e-cig use, female | Never/former e-cig use, female | aHR | 1.46 | 0.55–3.86 | Model 2 |
| Adebisi 2026 | Prevalent self-reported HTN | Former smokers | Never users | aOR | 1.10 | 1.02–1.20 | Fully adjusted logistic regression (Table S2) |
| Adebisi 2026 | Prevalent self-reported HTN | Current cigarette smoking only | Never users | aOR | 0.94 | 0.84–1.06 | Fully adjusted logistic regression |
| Adebisi 2026 | Prevalent self-reported HTN | Current e-cigarette use only | Never users | aOR | 0.77 | 0.63–0.93 | Fully adjusted logistic regression |
| Adebisi 2026 | Prevalent self-reported HTN | Current dual use | Never users | aOR | 0.95 | 0.75–1.20 | Fully adjusted logistic regression |
| Adebisi 2026 | Prevalent self-reported HTN | Exclusive e-cig users who were former smokers | Never users | aOR | 0.77 | 0.63–0.94 | Fully adjusted logistic regression |
| Adebisi 2026 | Prevalent self-reported HTN | Exclusive e-cig users who were never smokers | Never users | aOR | 0.74 | 0.34–1.62 | Fully adjusted logistic regression |
| Adebisi 2026 | Prevalent self-reported HTN | Current e-cigarette use only | Current cigarette smoking only | aOR | 0.81 | 0.66–0.99 | Fully adjusted logistic regression |
| Adebisi 2026 | Prevalent self-reported HTN | Current dual use | Current cigarette smoking only | aOR | 1.01 | 0.79–1.29 | Fully adjusted logistic regression |
| Adebisi 2026 | Prevalent self-reported HTN | Former smokers | Current cigarette smoking only | aOR | 1.17 | 1.04–1.32 | Fully adjusted logistic regression |
| Adebisi 2026 | Prevalent self-reported HTN | Never users | Current cigarette smoking only | aOR | 1.06 | 0.94–1.19 | Fully adjusted logistic regression |
| Corsi & Agbaje 2026 | Measured HTN | Any smoking and/or vaping | No smoking or vaping | aOR | 1.46 | 1.06–1.99 | Fully adjusted logistic regression (Table S2) |
| Corsi & Agbaje 2026 | Measured HTN | Exclusive smoker | No nicotine use | aOR | 1.51 | 1.07–2.12 | Fully adjusted logistic regression |
| Corsi & Agbaje 2026 | Measured HTN | Exclusive vaper | No nicotine use | aOR | 1.05 | 0.51–2.14 | Fully adjusted logistic regression |
| Corsi & Agbaje 2026 | Measured HTN | Dual user | No nicotine use | aOR | 1.57 | 0.77–3.20 | Fully adjusted logistic regression |

*HTN hypertension; aOR/AOR/OR odds ratio; aHR/HR hazard ratio; aPR adjusted prevalence ratio; ATET average treatment effect on the treated; PS propensity score; ENDS electronic nicotine delivery systems; BMI body-mass index; CI confidence interval. Estimates from the same cohort were not double-counted in any single pooled analysis: Shi et al. and Cook et al. both use PATH longitudinal data, so Cook was primary and Shi was reserved for sex-stratified/sensitivity synthesis. Kim et al. and Corsi & Agbaje are measured-hypertension studies and were handled accordingly.*

**Table S4.** Study-level estimates carried into each primary pooled analysis

| **Primary analysis** | **Study** | **Exposure** | **Comparator** | **Author-reported estimate** |
| --- | --- | --- | --- | --- |
| Prevalent hypertension: exclusive e-cigarette use | Falk 2022 | E-cigarette only users | Never users | OR 1.244 (1.048–1.477) |
| Prevalent hypertension: exclusive e-cigarette use | Kim 2022 (male) | E-cigarette only | Male non-smoker | OR 1.22 (1.02–1.48) |
| Prevalent hypertension: exclusive e-cigarette use | Kim 2022 (female) | E-cigarette only | Female non-smoker | OR 1.41 (0.74–2.70) |
| Prevalent hypertension: exclusive e-cigarette use | Lu 2024 | E-cigarette only use | Never cigarette use | AOR 1.16 (0.61–2.24) |
| Prevalent hypertension: exclusive e-cigarette use | Adebisi 2026 | Current e-cigarette use only | Never users | aOR 0.77 (0.63–0.93) |
| Prevalent hypertension: exclusive e-cigarette use | Corsi & Agbaje 2026 | Exclusive vaper | No nicotine use | aOR 1.05 (0.51–2.15) |
| Prevalent: dual use | Miller 2021 | Dual user | Never smoker | aOR 1.77 (1.32–2.39) |
| Prevalent: dual use | Falk 2022 | Dual users | Never users | OR 1.66 (1.519–1.814) |
| Prevalent: dual use | Kim 2022 (male) | Dual smoker | Male non-smoker | OR 1.24 (1.11–1.39) |
| Prevalent: dual use | Kim 2022 (female) | Dual smoker | Female non-smoker | OR 1.44 (0.96–2.16) |
| Prevalent: dual use | Lu 2024 | Dual use | Never cigarette use | AOR 1.83 (1.03–3.27) |
| Prevalent: dual use | Adebisi 2026 | Current dual use | Never users | aOR 0.95 (0.75–1.20) |
| Prevalent: dual use | Corsi & Agbaje 2026 | Dual user | No nicotine use | aOR 1.57 (0.77–3.20) |
| Incident: e-cigarette use | Cook 2023 | Exclusive ENDS use | Non-use | aHR 1.00 (0.68–1.47) |
| Incident: e-cigarette use | Erhabor 2025 | Exclusive e-cigarette use | Non-use | aHR 1.01 (0.83–1.23) |
| Incident: e-cigarette use | Lee 2026 | Current e-cigarette use | Never e-cigarette use | aHR 1.89 (1.12–3.18) |
| Incident: dual use | Cook 2023 | Dual use | Non-use | aHR 1.15 (0.87–1.52) |
| Incident: dual use | Erhabor 2025 | Dual use | Non-use | aHR 1.21 (1.10–1.34) |
| Incident: dual use | Lee 2026 | Dual ever use | Never users | aHR 1.85 (1.37–2.49) |

*AOR/aOR, adjusted odds ratio; ENDS, electronic nicotine delivery systems; HR/aHR, adjusted hazard ratio; OR, odds ratio. Lee et al. contributed the current e-cigarette-use estimate, rather than the ever-use estimate, to the incident e-cigarette-use pool to avoid within-study double-counting. Prevalent hypertension estimates reported as prevalence ratios were not pooled with odds ratios.*

**Table S5.** Study-level risk of bias (ROBINS-E)

| **Study** | **Overall judgement** | **Main reasons for the judgement** |
| --- | --- | --- |
| Miller 2021 | High | Cross-sectional temporality; self-reported outcome; residual smoking-history confounding |
| Falk 2022 | High | Cross-sectional temporality; self-reported outcome; limited adjustment set |
| Kim 2022 | High | Cross-sectional design; measured hypertension only among non-medicated participants; sex-stratified estimates |
| Shi 2023 | Some concerns | Self-reported outcome; short follow-up; limited power in e-cigarette strata; PATH overlap with Cook |
| Cook 2023 | Some concerns | Self-reported outcome; residual confounding by smoking history and health behaviour |
| Lu 2024 | High | Cross-sectional temporality; composite hypertension definition; residual confounding |
| Erhabor 2025 | Some concerns | Volunteer cohort/selection concerns; limited adjustment; EHR capture differences |
| Lee 2026 | Some concerns | Self-reported exposure; specific Hispanic/Latino cohort; residual confounding possible |
| Adebisi 2026 | High | Cross-sectional temporality; self-reported diagnosis; healthy-switcher bias |
| Corsi & Agbaje 2026 | High | Cross-sectional temporality; recent 5-day exposure window; sample includes adolescents |

*ROBINS-E Risk Of Bias In Non-randomized Studies – of Exposure; EHR electronic health record; PATH Population Assessment of Tobacco and Health. No study was judged to be at low overall risk of bias.*

**Table S6.** GRADE certainty of evidence — primary analyses (domain-level)

| **Comparison — outcome** | **Pooled effect** | **Risk of bias** | **Inconsistency** | **Indirectness** | **Imprecision** | **Publication bias** | **Certainty** |
| --- | --- | --- | --- | --- | --- | --- | --- |
| Exclusive e-cig use vs never/non-use — prevalent | OR 1.09 (0.88–1.36) | Serious | Serious (I²=68.1%) | Some concern | Serious; CI includes null | Not assessed (k<10) | Very low |
| Dual use vs never/non-use — prevalent | OR 1.41 (1.16–1.72) | Serious | Serious (I²=80.9%) | Some concern | Not serious; CI excludes null | Not assessed (k<10) | Very low |
| Exclusive e-cig use vs non-use — incident | HR 1.17 (0.83–1.64) | Some concerns | Serious (I²=64.3%) | Some concern | Serious; CI includes null | Not assessed (k<10) | Very low |
| Dual use vs non-use — incident | HR 1.35 (1.03–1.76) | Some concerns | Serious (I²=77.3%) | Some concern | Some concern; lower CI near null | Not assessed (k<10) | Very low |

*GRADE Grading of Recommendations Assessment, Development and Evaluation; OR odds ratio; HR hazard ratio; CI confidence interval; k number of estimates. Certainty began at low because all studies were observational and was downgraded for serious concerns.*

# Supplementary Figures

**Fig. S1. Forest plot of cigarette-only smoking versus never/non-use for prevalent hypertension.**

**Fig. S2. Forest plot of cigarette-only smoking versus non-use for incident hypertension.**

**Fig. S3. Forest plot of the former-smoker/current-vaper subgroup versus never/non-use for prevalent hypertension.**

**Fig. S4. Sensitivity forest plot of exclusive e-cigarette use versus never/non-use for prevalent diagnosed/self-reported hypertension.**

**Fig. S5. Sensitivity forest plot of dual use versus never/non-use for prevalent diagnosed/self-reported hypertension.**

# Fig. S6. Sensitivity forest plot of exclusive e-cigarette use versus non-use/never use for incident hypertension using Shi et al. instead of Cook et al. Cook et al. and Shi et al. both used PATH longitudinal data; therefore, Shi et al. was used only in sensitivity analysis to avoid double-counting the same cohort.

# Fig. S7. Sensitivity forest plot of dual use versus non-use/never use for incident hypertension using Shi et al. instead of Cook et al. Cook et al. and Shi et al. both used PATH longitudinal data; therefore, Shi et al. was used only in sensitivity analysis to avoid double-counting.
